# Pathologic motion patterns in patients with progressive pseudorheumatoid arthropathy of childhood

**DOI:** 10.1186/1546-0096-9-S1-P269

**Published:** 2011-09-14

**Authors:** M Hartmann, F Kreuzpointner, R Haefner, J-P Haas

**Affiliations:** 1 German Center for Pediatric and Adolescent Rheumatology, Garmisch-Partenkirchen; 2 Department of Biomechanics in Sports, Technische Universität München

## Introduction

Progressive pseudorheumatoid arthropathy of childhood (PPAC) is a specific subtype of spondyloepiphyseal dysplasie (SED) tarda. The skeletal disorder and is characterized by polyarthropathy of large and small joints. Typical signs are prominent epiphysis, progressive joint stiffness, muscle weakness and early fatigue [1]. Optimized physiotherapy is the only effective strategy to slow down the progress of functional disability.

## Aim

Quantification of pathologic motion patterns during walking.

## Methods

In a retrospective study eight adolescents suffering from PPAC (sex:female=1; male=7; age:14.6y; weight:50.0kg; size:1.42m; BMI:25) were compared with 20 healthy young person (cg) (sex:female=17, male=3; age:17.9y; weight:53.8kg; size:1.59m; BMI:21). 3D-gaitanalysis was performed with infrared cameras and the Plug-in-Gait Model. Analyses focused spatio-temporal and kinematic parameters in the sagittal plane. Mann-Whitney-U-Tests (p<0.05) and correlation calculations (Pearson) between age, body-mass-index (BMI), kinematic and spatio-temporal-parameters were used for statistical analysis.

## Results

Patients with PPAC walk very slow (p<0.001) with short step length (p<0.001) and broadened step width (p<0.001). The foot off occurs noticeable late (p<0.001). The kinematic data are highly significant different to the cg in pelvis, hip, knee and ankle. Especially the range of motion (ROM) in the hip, in the knee flexion (loading response) and extension (single support phase) as well as the ankle ROM (plantar flexion while push off) are decreased. Within the PPAC-group high negative correlations appear between BMI and ankle ROM (plantar flexion (push off)) (r=-.860).

**Table 1:** Selection of kenematic results in pelvis, knee and ankle joint.

	PPAC (n=8)		Control Group (n=20)		M.-W. –U-Test Sign. (2-tailed)
	
	Median	Q25/Q75	Median	Q25/Q75	p<0.01
Pelvic tilt	19.4	(15.1/21.6)	11.2	(8.5/12.8)	p<0.001

Hip ROM (Flex/Ext)	32.0	(28.3/34.6)	44.0	(42.0/46.4)	p<0.001

Knee ROM (Flex, loading response)	5.3	(4.4/6.9)	11.6	(10.7/13.4)	p<0.001

Knee ROM (Ext, single support1)	2.5	(1.1/4.7)	15.4	(13.6/17.8)	p<0.001

Ankle ROM (Plan-Flex, push off)	12.8	(11.3/13.0)	29.7	(26.8/35.5)	p<0.001

## Conclusion

The results are determined by very small ROM in the lower limb due to distinctive joint stiffness and muscle weakness. The effects of muscular weakness are intensified by high body weight.

The use of 3D-gaitanalysis is a helpful tool to individualize functional treatment to decelerate the progressive joint destruction in the lower limb.
